# Effects of Drying Methods on Serum Protein Powder Properties

**DOI:** 10.3390/foods11141996

**Published:** 2022-07-06

**Authors:** Jie Zhang, Yadi Li, Peng Zhou

**Affiliations:** 1 State Key Laboratory of Food Science and Technology, Jiangnan University, Wuxi 214122, China; jiezhang11@jiangnan.edu.cn; 2 International Joint Research Laboratory for Functional Dairy Protein Ingredients, U.S.-China Dairy Innovation Center, Jiangnan University, Wuxi 214122, China; 6210113050@stu.jiangnan.edu.cn

**Keywords:** serum protein concentrate, low-pressure spray drying, freeze drying, serum protein powder, proteomics

## Abstract

This study investigated the effects of atmospheric spray drying (ASD), low-pressure spray drying (LPSD) and freeze drying (FD) on the properties of serum protein powder, including the basic characteristics of the powder, bioactive proteins and changes in protein profile, using a proteomics approach. The total solid and water activity of the powder obtained by FD was significantly higher than that obtained by ASD and LPSD. There was no significant difference in the content of fat, lactose or solubility between the three kinds of powders. The concentration and activity of the proteins/enzymes in the serum protein powder made from LPSD were not altered after drying, similar to FD, although both parameters decreased after ASD. The microstructure of the powder observed by scanning electron microscopy indicated that the powder manufactured by ASD and LPSD was spherical in structure, while that manufactured by FD was flake-like. In total, there were 245 proteins identified in the serum protein concentrate and powder from the three drying methods. These findings indicate that LPSD is an effective and cost-saving method for producing serum whey protein powder.

## 1. Introduction

In the food industry, serum protein concentrate (SPC) and isolates are widely used food ingredients due to their high-quality nutrition and desirable functional properties. Infant formula is an important application of these components due to the low percentage of serum proteins (20%) in bovine milk protein, while this percentage is 60% in human milk [1]. Serum proteins are combined with bovine milk to increase their percentage in infant formula. Some serum proteins, such as immunoglobulin (Ig) G, IgA, IgM, lactoferrin (LF) and xanthine oxidase (XO), have been reported to show antibacterial, anti-pathogenic, anticarcinogenic and anti-inflammatory properties to protect infants against various infection [2,3].

The conventional manufacture of whey protein concentrate involves the by-products of cheese production, which can be separated into acid whey and sweet whey. During this process, heating is the main factor that can affect these functional proteins due to the thermal denaturation of the structure of proteins. Presently, microfiltration of skim milk to remove bacteria and to separate whey and casein has been widely used to obtain native serum proteins [4], and they are an excellent source of serum protein powder.

Powder is the preferred trade form due to its long shelf life and convenient handling. The commonly used technique for obtaining dairy powder is atmospheric spray drying (ASD), during which water is quickly evaporated with a high temperature and dry air. The advantage of this technique is that the processing time is short, although it may lead to the loss of heat-sensitive bioactive components [5]. Freeze drying (FD) is another drying method that is currently used, and it involves dehydration by sublimation. It can preserve bioactive components due to the low temperature (−80 °C). However, it is time-consuming and expensive [6]. Low-pressure spray drying (LPSD) processes samples at a low temperature and low pressure, which can prevent the denaturation of whey protein and the occurrence of the Maillard reaction. Thus, it is possible to prepare products that retain more active protein and have a longer shelf life to inhibit the growth of microorganisms [7].

To our knowledge, information related to the characteristics of serum protein powder prepared by microfiltration and different drying methods is scarce. Therefore, the aim of this study was to compare the basic characteristics of serum protein powder manufactured by ASP, LPSD and FD. The concentration of bioactive components in the powder was studied. The proteomics profiles of serum protein powder were also investigated. 

## 2. Materials and Methods

### 2.1. Manufacture of Serum Protein Powder

**Figure 1:**
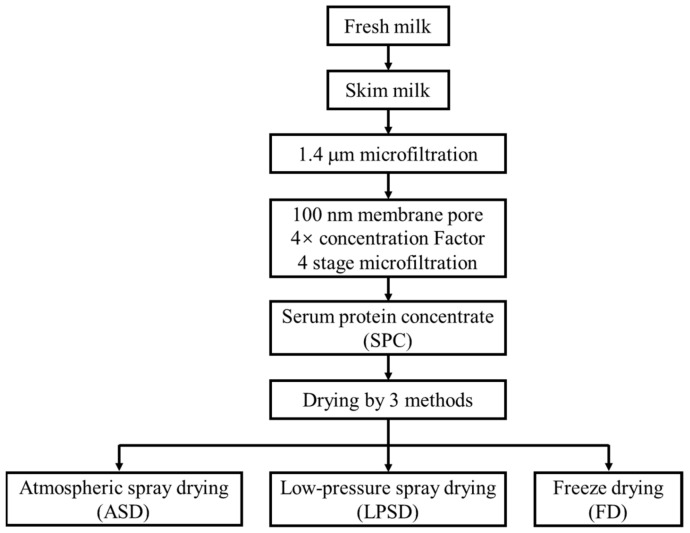
Schematic workflow of the preparation of the sample.

Fresh raw milk was purchased from Tianzi Dairy Co., Ltd. (Wuxi, Jiangsu, China). The fat, bacteria and somatic cells in the milk were removed using the same equipment and methods as described previously [4]. As shown in Figure 1, the obtained milk was subjected to microfiltration to separate serum proteins and casein using a 100 nm ceramic membrane at 50 °C, and 110 kPa with four concentration factors and four filtration stages (three diafiltrations). Next, the permeate obtained by microfiltration was concentrated using an ultrafiltration system with a membrane pore size of 10 kDa, during which diafiltration was performed three times to remove small molecules such as inorganic salts and lactose. The SPC obtained from microfiltration, which was composed of 10% solid matrix, was dried by ASD, LPSD and FD. The inlet temperature and outlet temperature for ASD were 185 °C and 85 °C, respectively, and it employed a B-290 spray dryer (BUCHI Labortechnik AG, Flawil, Switzerland), while they were 115 °C and 55 °C, respectively, at a pressure of 0.03 MPa for LPSD, which employed a YC-2000 (Shanghai Pilotech Instrument and Equipment Co., Ltd., Shanghai, China). FD was processed at −80 °C and 30 Pa, with drying for 72 h employing an Advantage EL-85 (SP Scientific, Inc. New York, NY, USA). The obtained serum protein powder was used for further analysis.

### 2.2. Microstructure of Serum Protein Powder

Serum protein powder was affixed on copper stubs and gold-coated. The surface of the serum protein powder was observed using an S-4700 scanning electron microscope (SEM, Hitachi Ltd., Tokyo, Japan) with a voltage of 15 kV.

### 2.3. Basic Characteristics of Serum Protein Powder

The content of total solids, fat and lactose in the serum protein powder was measured according to the method described by Deshwal et al. (2020). The protein content was analyzed by dissolving 1.00 g of serum protein powder in 9.00 g of ultrapure water and centrifuging at 700 g at room temperature for 10 min. The supernatant was collected and analyzed using the Bradford assay kit (Thermo Fisher Scientific, Waltham, MA, USA). The water activity was determined using a dew point activity meter (Novasina LabSwift-aw, Neuheimstrasse, Lachen, Switzerland). The serum protein powder was dissolved (10%, *w*/*w*) in ultrapure water under continuous stirring for 30 min. The solubility of the powder was measured and calculated as previously described [8].

### 2.4. Sodium Dodecyl Sulfate–Polyacrylamide Gel Electrophoresis (SDS-PAGE)

Sodium dodecyl sulfate–polyacrylamide gel electrophoresis was performed according to the method of Laemmli (1970) with a 4% stacking gel and a 13% resolving gel. The reconstituted powder (10%) was mixed with the sample buffer (Bio-Rad Laboratories, Inc., Hercules, CA, USA) containing 5% β-mercaptoethanol at a ratio of 1:1 (*v*/*v*). Next, 10 μL of sample solution was loaded into each well. Electrophoresis was performed with a Mini-PROTEAN Tetra Cell system (Bio-Rad Laboratories, Inc.). 

### 2.5. Protein Analysis by Enzyme-Linked Immunosorbent Assay (ELISA)

Concentrations of IgG, IgA, IgM and LF were determined using specific ELISA (Bethyl Laboratories, Inc., Montgomery, TX, USA) according to the manufacturer’s instructions. Samples were diluted by a factor between 2000 and 7000. The activity of XO was analyzed according to the methods of Zou et al. (2020).

### 2.6. LC-MS/MS Analysis

The reconstituted serum protein powder (10%) was subjected to protein digestion as previously described. Approximately 100 mg of protein was filtered using a 10-kDa Microcon unit (Millipore, Cork, Ireland). Next, 200 mL of 50 mM ammonium bicarbonate was added and centrifuged. Dithiotheritol was added at 20 mM and incubated for 30 min at 56 °C, and then 50 mM iodoacetamide was added and incubated for 30 min at 37 °C. Prechilled acetone was added, followed by incubation for 3 h at −20 °C, and the precipitate was collected and washed with acetone–water (6:1) after centrifugation. Trypsin was added to the protein sample at a mass ratio of 1:50, and the mixture was incubated overnight at 37 °C. The digested peptides were dried and resuspended in 50 mL of 0.1% formic acid, followed by desalting using a C18 column prior to LC-MS/MS analysis.

Approximately 10 mL of the digested solution was injected into a fused silica trap column (internal diameter, 100 μm id × 2 cm) packed in-house with reversed-phase silica (Reprosil-Pur C18AQ, 5 μm, Dr. Maisch GmbH) and analyzed using a Q-Exactive instrument (Thermo Finnigan) equipped with an Easy nano-LC 1000 HPLC system (Thermo Scientific). Mobile phase A was 0.1% formic acid in water, and mobile phase B was 0.1% formic acid in acetonitrile. The MS data were obtained with a mass range of 300 to 1600 *m*/*z*, resolution of 70,000, AGC target of 3e6 and maximum IT of 40 ms. Tandem MS was set at a resolution of 17,500, AGC target of 1e5 and maximum IT of 60 ms.

### 2.7. Statistical Analysis 

The experiments were conducted three independent times, and data were analyzed using IBM SPSS Statistics 25 (IBM SPSS Statistics for Windows, IBM Corp., Armonk, NY, USA). The Duncan test was used to assess the difference between the means, and *p* < 0.05 was considered as statistically significant.

## 3. Results and Discussion

### 3.1. Physiochemical Properties of Serum Protein Powder

**Figure 2:**
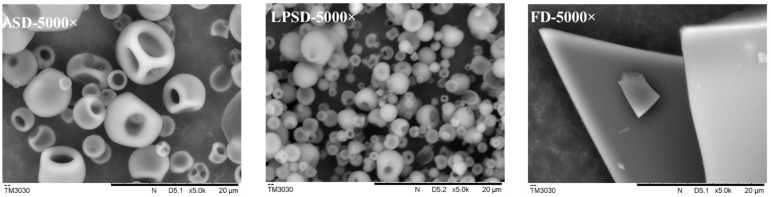
Surface microstructure of serum protein powder prepared by ASD, LPSD and FD.

The microstructure of serum protein powder produced by different drying methods was observed by scanning electron microscopy (Figure 2). The morphological characteristics of serum protein powder prepared by different drying methods varied greatly. The powder prepared by ASD and LPSD exhibited spherical structures, but the LPSD sample had more spherical structures and less surface depressions than that prepared by ASD, which may be related to the lower pressure. The structure of FD samples was flake-like, which may be related to the drying process. The moisture in the FD sample was directly sublimated from the solid state to the gaseous state, so that water molecules were transferred from the interior of the sample to the surface of the sample, and then rapidly sublimated. However, ASD tended to shrink the particles during water vaporization, which maintained the shape of particles after drying [9]. These observations are consistent with those for the powder prepared from camel milk [7]. 

The serum protein concentration obtained by microfiltration was dried by ASD, LPSD and FD. The basic characteristics, including the total solid content, water activity, fat content, lactose and protein and solubility, of the serum protein powder are shown in Table 1. The powder obtained by FD had a significantly higher total solid content than those obtained by ASD and LPSD, which were similar to each other. FD could remove the water in powder to achieve a very low level, approximately 2%, which is consistent with a previous study [7]. This may be due to the long processing time of FD, which allowed it sufficient time to remove water. The water activity of the powder produced by LPSD (0.277) was higher than that of the power produced by ASD (0.169) and FD (0.036). The water activity represented the extent of water removal and the equilibrium conditions at the end of the drying process. The content of fat was not detectable for any of the three types of powder, due to that fact that the liquid serum protein concentrate used for drying was made from skim milk and subjected to 100 nm microfiltration, which prevented the fat globules from passing through. The low lactose content (<2%) was due to the diafiltration process during the manufacture of liquid whey protein concentrate, which removed lactose. There was no significant difference between the solubility of the powders prepared by the three drying methods, which all dissolved well in water.

### 3.2. Electrophoresis of Serum Protein Powders

**Figure 3:**
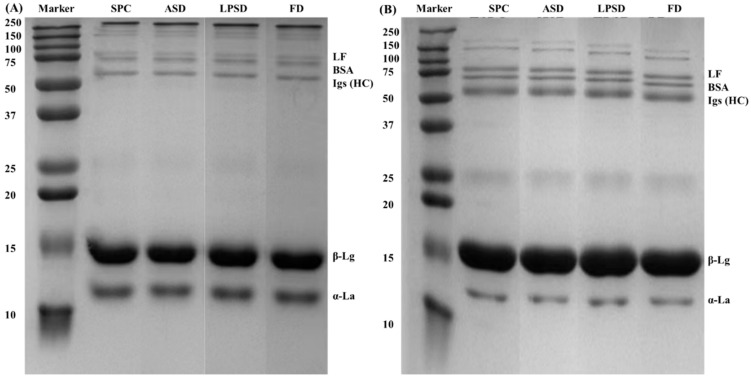
Non-reducing (**A**) and reducing SDS-PAGE (**B**) of serum protein profiles of SPC, ASD, LPSD and FD.

Figure 3 shows the protein profiles of the serum protein powder and SPC observed by reducing and non-reducing SDS-PAGE. β-lactoglobulin and α-lactalbumin were the main proteins found in all samples, as well as lactoferrin, bovine serum albumin and the immunoglobulin heavy chain. However, there was no difference in protein composition and concentration among these samples that could be detected via SDS-PAGE. A band corresponding to a molecular weight greater than 250 kDa in non-reducing SDS-PAGE suggested polymer formation. No casein band was observed in any of the three samples, which indicated a good separation of casein and serum.

### 3.3. Concentrations of Proteins in Serum Protein Powder

**Figure 4:**
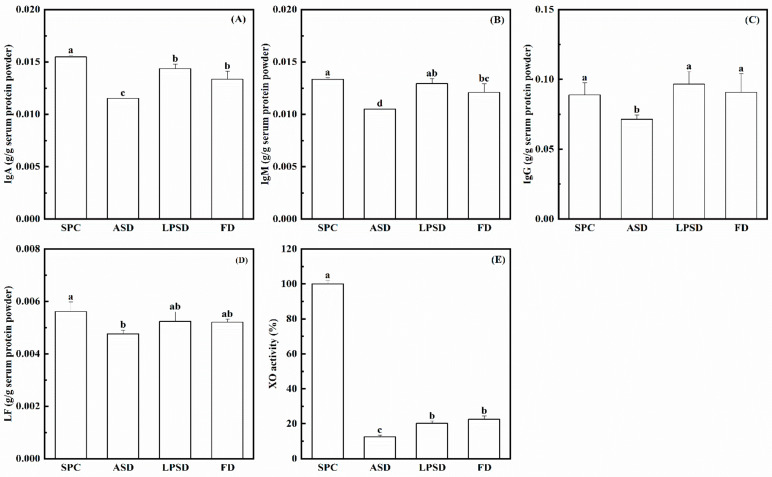
The contents of native (**A**) IgA, (**B**) IgM, (**C**) IgG, (**D**) LF and (**E**) bioactive XO in serum protein powder prepared by different drying methods. Different letters indicate significant difference (*p* < 0.05).

In industry, the main drying method to prepare liquid serum protein concentrate is ASD, which also undergoes a certain degree of thermal denaturation during the drying process. LPSD allows the liquid to be dried at relatively low temperatures due to its lower pressure within the spray drying equipment during vacuuming. Due to its lower drying temperature, denaturation of whey proteins, especially low-abundance biologically active proteins, can be avoided, which protects the biological function of these components in serum proteins. The concentrations of individual proteins determined by ELISA in the serum protein powder prepared by ASD, LPSD and FD are shown in Figure 4. As can be calculated from the total serum protein and the individual proteins in the previous review, the contents of IgA, IgM, IgG and LF were in the range of 0.0214–0.1450, 0.0071–0.0254, 0.0028–0.0909 and 0.0057–0.0181 g/g serum protein, respectively [10]. The four proteins measured in this study were within these ranges. After the liquid serum protein concentrate was dried by LPSD, the contents of IgG, IgM and LF were not found to be significantly different from those before drying, indicating that LPSD did not affect these bioactive proteins. Compared with FD, there was no statistical difference in the content of the three bioactive proteins in the powder prepared by LPSD. However, LPSD had a much higher efficiency than FD, as LPSD only takes several hours, as compared to FD, which takes several days. Thus, LPSD is a better choice for the manufacture of serum powders. The reason why the effects of LPSD and FD were consistent may be related to the drying temperature. Although the temperature of LPSD was much higher than that of FD, the temperature of LPSD was lower than the temperature at which the three proteins were denatured. The contents of IgA, IgM, IgG and LF in the serum protein powder prepared by FD decreased by 25.8%, 21.1% 20.2% and 15.2%, respectively, compared with the liquid serum protein concentrate, indicating that these proteins were partially inactivated by ASD. The effect of ASD on active proteins was consistent with the results of a previous study using ASD to treat fresh milk [11]. The contents of these proteins in the commercial whey protein powder were also measured, and they were much lower than those in the serum protein powder prepared by ASD, LPSD and FD (data not shown). This indicates that the liquid serum protein concentrate obtained from membrane filtration can protect these proteins in milk. The relative enzyme activity of XO decreased by more than 80% after drying, with the lowest decrease in ASD powder. There was no significant difference in XO activity between the serum protein powder prepared by LPSD and FD, while ASD significantly reduced XO activity compared with the other two methods. In summary, LPSD can preserve the concentration of proteins and activity of enzymes to the same extent as FD, while LPSD had a much higher production efficiency.

### 3.4. Proteomics

**Figure 5:**
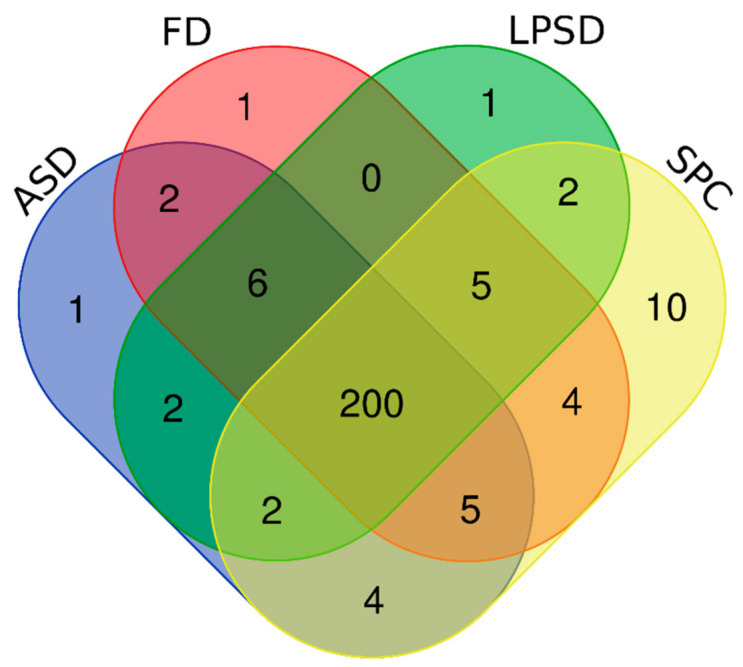
Venn diagram of the identified serum proteins from SPC, ASD, LPSD and FD.

The Venn diagram of the identified proteins from SPC, ASP, LPSD and FD powders is shown in Figure 5. A total of 245 proteins were identified in these four groups by peptide identification, with a total of 200 proteins in all groups. There were 232, 222, 218 and 223 proteins identified in SPC, ASD, LPSD and FD powder samples, respectively. Compared with SPC, only 21, 23 and 18 proteins were not identified in ASD, LPSD and FD powders, respectively. The high similarities between different powder samples may have resulted from the fact that water removal was the main activity during drying, which may not affect the protein profile. However, some difference was observed, which may be due to the change in protein structure under different processing temperatures. Whey protein concentrate was demonstrated to have 192 proteins, which was lower than that observed for SPC [12]. This discrepancy may be due to the fact that we used microfiltration, while they used centrifugation and ultracentrifugation to separate milk fat globule membrane proteins and casein. A total of 162 proteins were identified in a commercially available whey protein concentrate or whey protein isolated from bovine milk in a previous study [13]. The difference in the number of identified proteins may be due to the different manufacturing procedures for whey protein powder as well as the higher sensitivity of our method [12].

**Figure 6:**
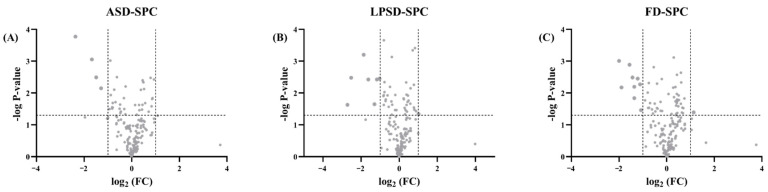
Volcano plots of serum proteins after different drying methods compared with SPC. (**A**) Represents the significantly changed proteins in ASD powder compared with SPC. (**B**) Represents the significantly changed proteins in LPSD powder compared with SPC. (**C**) Represents the significantly changed proteins in FD compared with SPC.

Proteins with significant changes between the four groups are illustrated by volcano plots, as shown in Figure 6. The significance level was selected based on a fold change > 2 or <2 and *p* < 0.05. Based on these levels, there were 4, 8 and 10 proteins that were significantly changed after ASD, LPSD and FD, respectively. Among them, three proteins, namely 45-kDa calcium-binding protein, fibrinogen gamma-B chain and TKT protein, increased in the three drying methods (Table S1). Liu, Zhang [11] also reported the increased abundance of 45-kDa calcium-binding protein after heating and ASD, which is consistent with the data obtained here. It was reported that 45-kDa calcium-binding protein was a complex containing 8-kDa calcium-binding protein dependent on calcium and disulfide bond formation [14]. Therefore, the increased abundance of this protein may have resulted from drying. TKT was reported to have several functions, such as calcium and magnesium ion binding, protein homodimerization and thiamine pyrophosphate binding. The content of TKT was significantly higher in mature milk than in transitional milk [15]. The other increased protein in ASD powder was the renin receptor, whose content was also increased after FD. This protein plays a vital role in the renin–angiotensin system, which is associated with hypertension and diabetes [16]. For LPSD, the eight significantly changed proteins were also changed in FD samples, and the other five proteins were either decreased (apolipoprotein C-III) or increased (endoplasmic reticulum chaperone BiP, cathelicidin 1, protein OS-9 and haptoglobin). Apolipoprotein C-III binds to the high-density lipoprotein particle receptor, while phospholipid functions as a lipase inhibitor, which is very important for the homeostasis of triglyceride levels [17]. The endoplasmic reticulum chaperone BiP is essential for the cytoplasmic assembly compartment, and its depletion results in the loss of lamin phosphorylation [18]. Cathelicidins possess antimicrobial activity and promote inflammation, and their abundance is increased in mastitic cow milk [19,20]. The only protein that changed in FD powder, and not in the other two types of powder, was nucleoside diphosphate kinase B. This protein functions as a regulator of endocytosis and tracheal development [21].

## 4. Conclusions

The effects of ASD, LPSD and FD on the microstructure, basic characteristics and protein profiles of serum protein powder were studied. The powder prepared by LPSD had fewer surface depressions than that prepared by ASD, although the structure of both were spherical. However, the powder prepared by FD had a flake-like structure. The powder obtained by FD had a higher solid content, lower water content and lower water activity than those obtained via the other two methods. No fat was detected in any of the three samples, and there was no difference between the solubility of these three. The protein profile revealed by SDS-PAGE indicated no difference between three samples. The content of native proteins and the activity of XO in LPSD powder were similar to those in FD powder. Although no significant difference in the protein profiles was observed in SDS-PAGE, proteomics analysis indicated differences between the three samples.

## Figures and Tables

**Table 1:** Basic characteristics of serum protein powder manufactured by ASD, LPSD and FD.

Sample	ASD	LPSD	FD
Total solids	95.8% ± 0.1% ^b^	95.0% ± 1.9% ^b^	98.2% ± 1.9% ^a^
Solubility (%)	98.3 ± 0.6 ^a^	98.6 ± 1.8 ^a^	98.1 ± 0.7 ^a^
Water activity	0.169 ± 0.004 ^b^	0.277 ± 0.007 ^a^	0.036 ± 0.038 ^c^
Fat	ND	ND	ND
Lactose	<2%	<2%	<2%

Note: means in a row followed by different lower-case letters differ significantly (*p* < 0.05).

## Data Availability

Data is contained within the article or Supplementary Material.

## Supplementary Materials

The following supporting information can be downloaded at: https://www.mdpi.com/article/10.3390/foods11141996/s1. Table S1: List of significantly changed proteins in atmospheric spray drying (ASD), low-pressure spray drying (LPSD) and freeze drying (FD) compared to serum protein concentrate (SPC).
