# Supplementary material for: Effects of Drying Methods on Serum Protein Powder Properties
Source: Foods. 2022 Jul 6;11(14):1996. doi: 10.3390/foods11141996 (PMC9317665; doi:10.3390/foods11141996)
Supplement: Supplementary file 1 [file foods-11-01996-s001.zip › foods-1795000-supplementary.pdf]

Supplementary

Table S1. List of significantly changed proteins in atmospheric spray drying (ASD), low-pressure spray drying (LPSD) and freeze drying (FD) compared to serum protein concentrate (SPC).

Samples	Entry name	Protein name	Gene
ASD-SPC	F1MKI5	45 kDa calcium-binding protein	SDF4
	A0A3Q1MV91	Fibrinogen gamma-B chain	FGG
	P81134	Renin receptor	ATP6AP2
	A7Z014	TKT protein	TKT
FD-SPC	P19035	Apolipoprotein C-III	APOC3
	Q0VCX2	Endoplasmic reticulum chaperone BiP	HSPA5
	A0A3Q1MV91	Fibrinogen gamma-B chain	FGG
	P81134	Renin receptor	ATP6AP2
	A7Z014	TKT protein	TKT
	Q3T0Q4	Nucleoside diphosphate kinase B	NME2
	Q3MHX6	Protein OS-9	OS9
	Q7M365	Haptoglobin	NA
	A0A3Q1LRG2	Cathelicidin 1	LOC112441458
LPSD-SPC	P19035	Apolipoprotein C-III	APOC3
	Q0VCX2	Endoplasmic reticulum chaperone BiP	HSPA5
	F1MKI5	45 kDa calcium-binding protein	SDF4
	A0A3Q1MV91	Fibrinogen gamma-B chain	FGG
	A7Z014	TKT protein	TKT
	A0A3Q1LRG2	Cathelicidin 1	LOC112441458
	Q3MHX6	Protein OS-9	OS9
	Q7M365	Haptoglobin	NA

NA: not available.
